# Mindfulness and sustainable diets: a meta-analysis and CO_2_ emission savings scenarios

**DOI:** 10.1186/s12937-026-01356-0

**Published:** 2026-07-06

**Authors:** Anna Kosteletzky, Stephanie Margarete Thomas, Carmen Jochem

**Affiliations:** 1 Department of Biogeography, University of Bayreuth, Bayreuth, Germany; 2 Bayreuth Center of Ecology and Environmental Research BayCEER, Bayreuth, Germany; 3 Chair of Planetary & Public Health, University of Bayreuth, Bayreuth, Germany

**Keywords:** Mindfulness, Sustainable diets, Planetary health, Meat consumption, Climate change mitigation

## Abstract

**Background:**

Dietary habits are a major driver of both human health outcomes and global greenhouse gas emissions, with excessive meat consumption being a key contributor. Mindfulness, understood as purposeful and non-judgmental awareness of the present moment, has been suggested as a psychological factor that can foster more sustainable food choices. However, the empirical evidence on the link between mindfulness and sustainable diets remains fragmented.

**Main body:**

This study presents the first systematic review and meta-analysis of the association between mindfulness and sustainable dietary behaviors. Twelve articles with 13 studies, published between 2018 and 2025, were included, covering both observational and interventional designs with sample sizes ranging from 16 to 560 participants. A random-effects model revealed a small but significant positive association between mindfulness and sustainable diets (d = 0.28, 95% CI [0.13, 0.43]). Subgroup analyses indicated that observational studies tended to show stronger effects than interventions, and that cultural context played an important moderating role, with larger effects reported in Asian samples. Facet-specific analyses suggested that certain mindfulness dimensions – particularly observing, describing, and non-reactivity – were more strongly associated with sustainable food choices, while others showed weaker or non-significant links. A focused analysis on meat consumption revealed that mindfulness was positively associated with reduced meat intake or vegetarian dietary intentions (d = 0.25). Scenario modelling using national Life Cycle Assessment data from Germany, the UK, and the U.S. demonstrated that the potential reductions in meat intake could translate into substantial CO_2_ emission savings. A full adoption of the EAT-Lancet diet was projected to result in cumulative savings of up to 11% of Germany’s total emissions budget by 2040, whereas incorporating the population’s willingness to reduce meat intake yielded more conservative cumulative savings of 2.8% over the same period.

**Conclusion:**

This meta-analysis provides systematic evidence that mindfulness is positively associated with more sustainable dietary behavior and reduced meat consumption. While effects are modest and heterogeneous, the findings suggest that mindfulness could represent a promising lever for promoting dietary change aligned with planetary health goals. Future research should further investigate cultural differences, the role of specific mindfulness facets, and the feasibility of scaling mindfulness-based interventions to support both individual well-being and climate targets.

**Supplementary Information:**

The online version contains supplementary material available at 10.1186/s12937-026-01356-0.

## Introduction

Climate change presents an urgent global challenge, significantly impacting ecosystems, food security, and human health [1, 2]. Food systems are among the largest contributors to greenhouse gas (GHG) emissions worldwide, with livestock production playing a particularly critical role [3–5]. Simultaneously, excessive consumption of resource-intensive, animal-based foods is linked to chronic diseases [6]. A shift toward sustainable dietary practices, including reduced meat consumption, is essential to mitigate climate and health impacts [7]. However, dietary behaviors are deeply embedded in social norms and individual habits, which often resist change.

Pro-environmental behavior (PEB), especially in nutrition, can support systemic transformation. A growing body of research suggests that psychological traits such as mindfulness may be pivotal in promoting sustainable dietary choices [8, 9]. Mindfulness – defined as purposeful, present-moment, non-judgmental awareness – has evolved into a secular tool for promoting wellbeing, stress reduction, and behavior change [10]. Several instruments capture mindfulness, most commonly the Comprehensive Inventory of Mindfulness Experiences (CHIME) and the Five Facet Mindfulness Questionnaire (FFMQ) are used for assessment. While the CHIME questionnaire includes eight subscales, the FFMQ assesses five mindfulness facets – observing, non-reactivity to internal experience, acting with awareness, non-judging of internal experience, and describing [11, 12]. Observing denotes the capacity to deliberately attend to and notice both internal and external experiences, including sensory perceptions, bodily sensations, thoughts, and emotions. Describing refers to the ability to articulate internal experiences by labeling them with appropriate verbal expressions. Acting with awareness involves engaging fully in ongoing activities with conscious attention, rather than functioning automatically. Non-judging of inner experience describes an attitude of refraining from evaluative appraisals of one’s thoughts and emotions, whereas Non-reactivity to inner experience reflects the tendency to permit thoughts and feelings to arise and pass without becoming entangled in or driven by them [13]. Previous studies suggest that specific facets of mindfulness, such as observing and non-reactivity, may foster pro-environmental behavior, whereas other facets, including non-judging, have been discussed as potentially neutral or even inhibiting behavioral change [9, 14]. Scientific interest in mindfulness has increased, with evidence pointing to its potential to interrupt automatic behaviors, bridge the attitude-behavior gap, and strengthen values like compassion and connectedness to nature – factors associated with PEB [9, 15]. Mindfulness interventions such as MBSR (Mindfulness-Based Stress Reduction) and its climate-focused adaptations (e.g., Mindful Eco Wellness) have shown promising results in improving awareness and aligning actions with pro-social and ecological values [8, 9, 16]. In the context of food-related greenhouse gas emissions, such alignment may translate into multiple dietary pathways, including reduced consumption of animal-based products as well as increased preference for organic, locally produced, and seasonal foods and potentially reduced food waste [17–20]. Mindfulness may support the interruption of habitual routines, thereby facilitating a shift from thoughtless consumer behavior toward more mindful consumption patterns [9, 15, 21–24]. Moreover, mindfulness has been shown to help bridge the attitude–behavior gap, enabling individuals to align their values and intentions more closely with actual behavior [8, 9, 15, 16, 22, 25]. In addition, mindfulness may promote altruistic values such as prosociality, connectedness to nature, compassion, and empathy. These value orientations can attenuate self-focused and egoistic tendencies, thereby fostering pro-ecological intentions and behaviors [9, 16, 26–29]. Beyond its environmental implications, mindfulness is also associated with improved personal health and well-being, including better sleep quality and reductions in stress, depressive symptoms, and physical pain [9, 16, 28, 30, 31].Yet, the empirical link between mindfulness and sustainable diet remains underexplored and inconsistent. Several studies report associations with lower meat consumption and more conscious food choices as was recently summarized in a scoping review [32]. Accordingly, Pompili & Carfora [32] found that mindful eating was linked to greater intake of plant-based, organic, local, and seasonal foods, higher adherence to the Mediterranean diet, and intentions to reduce animal-protein consumption, with only one study reporting a barrier regarding insect-based foods. Nonetheless, methodological heterogeneity and limited sample sizes challenge generalizability. Moreover, the potential CO_2_ savings from diet shifts driven by mindfulness are not yet quantified.

To our knowledge, the current study presents the first systematic review and meta-analysis to quantify the association between mindfulness and sustainable dietary behavior. It aims to synthesize findings from both observational and interventional studies, assess the role of different mindfulness dimensions, and explore subgroup differences across cultural and regional contexts. Moreover, we compute potential CO_2_ reductions based on EAT-Lancet dietary recommendations and varying levels of public willingness to reduce meat consumption in Germany, the UK, and the U.S. Overall, this study aims to enhance understanding of how mindfulness relates to dietary behavior, in order to inform interventions that promote both individual well-being and planetary health.

## Methods

### Systematic review and meta-analysis

The systematic review and meta-analysis aimed to investigate the relationship between mindfulness and sustainable dietary behavior and was conducted with methodological orientation based on the updated 2020 guidelines for reporting systematic reviews (PRISMA; Supplemental Table S1) [33]. The study protocol was registered a priori at PROSPERO under the ID CRD42023446703 (https://www.crd.york.ac.uk/PROSPERO/view/CRD42023446703 ). Eligible studies included both observational and intervention-based research designs, provided they examined how mindfulness or mindfulness-based interventions (e.g., MBSR) influence dietary behavior relevant to sustainability. Articles were included if they were peer-reviewed, available in English, published before January 15th, 2025, and provided sufficient data for effect size estimation based on validated outcome measures related to sustainable diets. Where the required information was not directly available, the corresponding authors were contacted to obtain the necessary data. Studies that solely examined weight loss, general health diets, or broader pro-environmental behaviors without explicit dietary items were excluded.

### Search strategy and study selection

A systematic search was conducted in the databases PubMed and Web of Science between December 21st, 2024, and January 15th, 2025. The search strategy targeted three thematic areas: mindfulness (e.g., “mindful*,” “MBSR,” “meditat*”), sustainability (e.g., “sustainab*,” “ecologic*,” “environment*”), and diet (e.g., “food,” “nutrition,” “eating”). To ensure specificity, all search terms were restricted to the title and abstract fields. Supplemental table S2 includes the full search term. Literature research was conducted in duplicate by A.K. and C.J. Results were screened independently by A.K. and C.J. for titles and abstracts, and full texts were assessed for eligibility if deemed relevant. Any discrepancies during this step were resolved through consensus. Duplicate records were manually identified and removed. Decisions regarding the inclusion of full-text papers were discussed between A.K. and C.J., and any discrepancies were resolved through consensus.

### Data extraction and coding

Data extraction was performed by A.K., focusing on the following characteristics: author name, publication year, study name and design, geographic region, sample size, participant age and sex distribution, follow-up period, methods of mindfulness assessments, effect estimates, reported sustainable dietary behaviour. Data extraction was carried out in duplicate by A.M. and C.J., and any inconsistencies were resolved through discussion with S.T.

Data extraction prioritized behavioral outcomes over attitudinal measures due to the well-documented attitude-behavior gap. For each study, one effect size (Cohen’s d) was computed to ensure statistical independence, with exceptions for studies containing independent subsamples. Depending on the statistical data available (e.g., t-values, correlations, ANOVAs), various standard conversion formulas were used. In cases reporting Pearson’s r or standardized β-coefficients, these were transformed into Cohen’s d using established methods [34, 35].

For intervention studies, only post-intervention data were included to avoid confounding from time-delayed behavioral changes or dropout effects. Within-subject and between-subject designs were accommodated, applying appropriate formulas for variance and pooled standard deviations. In cases where mindfulness was operationalized via multiple subscales, averages were computed based on Fisher’s z-transformation to preserve statistical validity.

### Statistical analysis for meta-analytic approach

All analyses were conducted using R (version 4.3.2), primarily through the metafor package. A random-effects model was applied, reflecting expected heterogeneity in study populations, mindfulness measures, and intervention designs. Heterogeneity was assessed using Cochran’s Q, I^2^, and τ^2^ statistics. Publication bias was evaluated via funnel plots, Egger’s regression, and the trim-and-fill method.

Sensitivity analyses were conducted using leave-one-out procedures and influence diagnostics (rstudent, Cook’s distance, DFFITS, covariance ratios, tau^2^ change, QE change, study influence due to predictor values and the weight a study contributes to the meta-analysis based on its variance) to identify high-impact studies. Additionally, robustness was tested with a Hartung-Knapp adjustment, which offers more conservative confidence intervals in the presence of heterogeneity.

### Subgroup analyses

To explore sources of heterogeneity, subgroup analyses were conducted based on study design (intervention vs. observational), region (Germany, UK, US, others), and type of mindfulness instrument used. Separate analyses were also conducted for specific dietary behaviors, such as meat consumption, and different dimensions of mindfulness as assessed by validated instruments like the CHIME and FFMQ.

### Environmental impact scenarios

To contextualize the potential climate impact of mindfulness-related reductions in meat consumption, we developed emission scenarios for those dietary behaviors that emerged as central outcomes in the quantitative synthesis: (a) Business-as-Usual (BAU), (b) Realistic Reduction (REDUCTION), and (c) Lancet Reference Diet (LANCET). Life Cycle Assessment (LCA) data from Clune et al. [36] and population-level consumption data for Germany, the UK, and the U.S. were used [37–39].The scenarios therefore translate the empirically observed associations between mindfulness and dietary behavior into estimates of CO_2_-equivalent emissions at the population level, using independent life-cycle assessment data and country-specific consumption patterns. The BAU scenario reflects current per-capita consumption of beef, pork, and poultry and serves as the baseline.

The LANCET scenario represents an idealized sustainable diet following the EAT-Lancet recommendations, with red meat intake set to 14 g/day and poultry to 29 g/day. Red meat intake was allocated to beef and pork according to national consumption ratios observed under BAU conditions. This daily intake was then extrapolated to an annual amount (365 days) resulting in 5.11 kg of red meat per person per year which was then multiplied by the regional proportions of beef and pork consumption. For the U.S., this corresponded to 2.25 kg of pork and 2.86 kg of beef per person annually under the LANCET scenario, assuming the relative distribution of red meat types remained unchanged. In Germany, the calculation yielded an annual consumption of 3.76 kg of pork and 1.35 kg of beef per person. In the UK, this translated to 3.09 kg of pork and 2.02 kg of beef per person per year. For poultry, the Lancet diet recommends resulted in an annual consumption of 10.585 kg per person across all regions.

The REDUCTION scenario captures a more behaviorally realistic pathway by incorporating country-specific survey data on willingness to reduce meat consumption. The Lancet reference diet was applied only to the population share willing to reduce meat consumption (35% in the U.S., 42% in Germany, and 61% in the UK) [40–43]; the remainder followed BAU patterns.

Emissions were calculated separately for each type of meat and aggregated using population-weighted averages. Emissions were calculated per capita and in total, for the current situation and projected to 2040 using official population data and dietary change assumptions based on national willingness-to-change surveys [40–43]. These results were then compared to national emissions budgets and climate targets.

## Results

### Overview of Included Studies

**Fig. 1:**
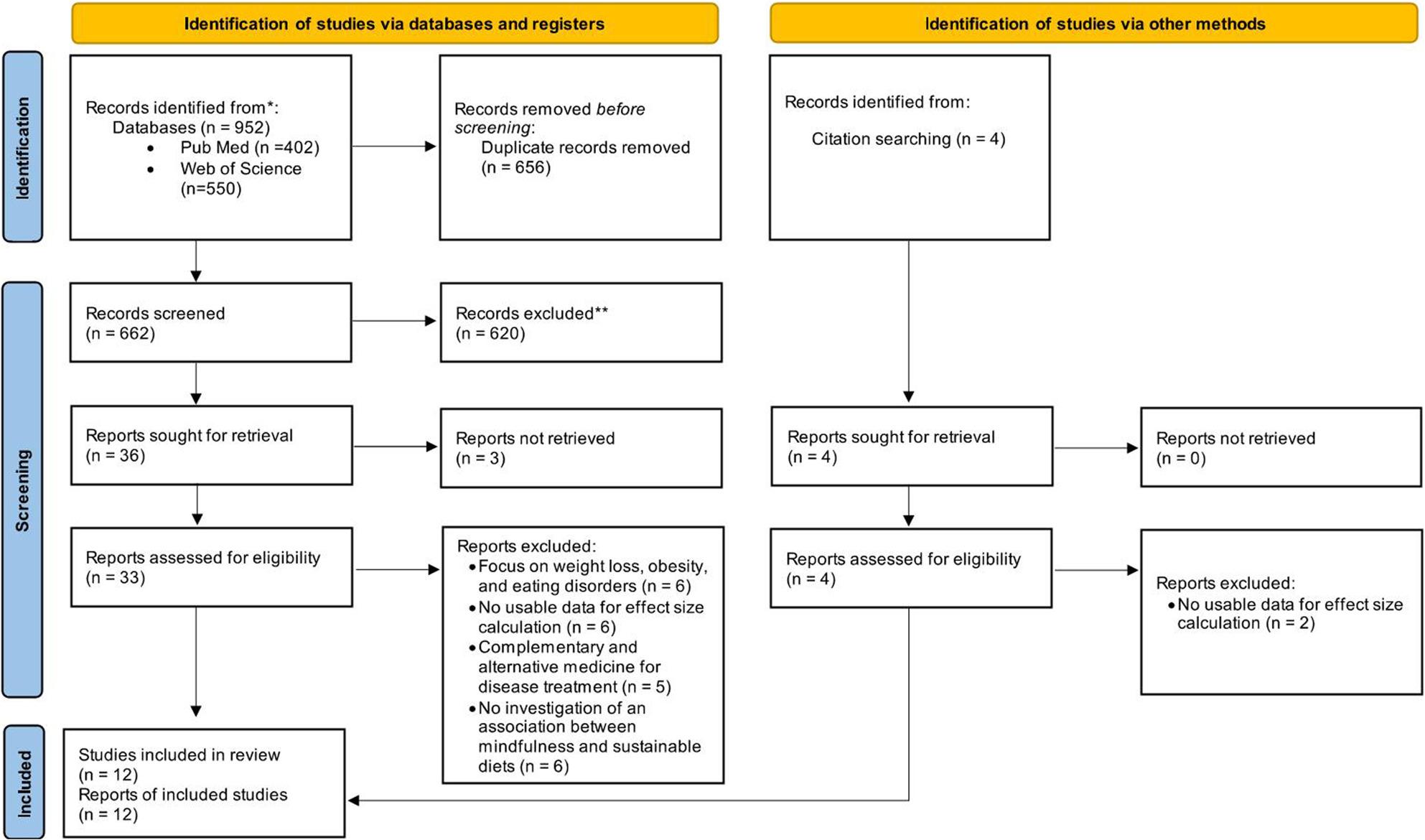
Flow chart following the Preferred Reporting Items for Systematic Reviews and Meta-Analyses (PRISMA) guidelines [33]. The search was conducted on PubMed and Web of Science. Finally, twelve papers were eligible for inclusion

Twelve articles, comprising a total of thirteen study records were included in the meta-analysis, as depicted in Fig. 1; Table 1 [17, 20, 22, 44–52]. The initial search in PubMed yielded 402 articles, while Web of Science returned 550 articles that matched the search criteria. Following the exclusion of duplicates from the databases, 254 records remained for screening from the Web of Science list. 

**Table 1:** Overview of included studies in the meta-analysis including study design, intervention type, measurement scales of mindfulness and sustainable diets, intervention duration, and number of participants [17, 20, 22, 44–52]

Study	Study design	Intervention type	Mindfulness Measurement	Sustainable Diet Measurement	Duration (weeks)	Number of participants	Effect sizes
Barett et al. (2024) [44]	Interventional study	Mindful Eco-Wellness (based on MBSR)	PROMIS-29	PEB with separate items on nutrition	7	16–19	Mean_Pre_ = 3.00, SD_Pre_ = 1.03, Mean_Post_ = 3.06, SD_Post_ = 0.94
Böhme et al. (2018) [45]	Interventional study	BiNKA Training (based on MBSR)	CHIME-A	Young consumers’ sustainable consumption behavior Scale	8	70 (IG:39, CG:31)	Mean_Pre, IG_ = 2.94, SD_Pre, IG_ = 0.77, Mean_Post, IG_ = 3.18, SD_Post, IG_ = 0.77; Mean_Pre, CG_ = 3.14, SD_Pre, CG_ = 0.93, Mean_Post, CG_ = 3.08, SD_Post, IG_ = 0.88
Geiger et al. (2019) [22]	Interventional study	sMBI (MBSR with few exercises on SCB)	CHIME	Cube Model of SCB (Nutrition)	8	131 (IG:58, CG:73)	F = 1.8
Hunecke & Richter (2019) [17]	Observational study	Not applicable	FFMQ	Five items (relating to animal-derived foods, preference for organic, local, and seasonal foods)	Not applicable	310	*r* = .147
Kumar & Panda (2025) [46]	Observational study	Not applicable	CHIME	Cube model of SCB (Nutrition)	Not applicable	519	*r* = .423
Ramstetter et al. (2023) [47]	Intervention	EU Climate Leadership Program	FFMQ	Four dimensions of PEB with items on nutrition	10	94 (IG:65, CG:29)	t = 0.459
Richter & Hunecke (2020) [20]	Observational study	Not applicable	FFMQ	Seven items (Organic food consumption, covering different types of food)	Not applicable	560	*r* = .11
Stanszus et al. (2019) [48]	Interventional study	MBI (Based on MBSR)	CHIME	Sustainable Consumption Behavior Nutrition Scale	8	76 (IG:37, CG:39)	Mean_Pre, IG_ = 3.50, SD_Pre, IG_ = 1.72, Mean_Post, IG_ = 3.55, SD_Post, IG_ = 1.73; Mean_Pre, CG_ = 3.56, SD_Pre, CG_ = 1.75, Mean_Post, CG_ = 3.64, SD_Post, IG_ = 1.67
Thiermann et al. (2020) [49]	Observational study	Not applicable	CHIME	Environmental impact of different practitioner levels	Not applicable	300	Odds ratio = 0.409, CI [0.231, 0.724]
Werner et al. (2020) [50]	Observational study	Not applicable	CHIME	PEB scale with items on nutrition	Not applicable	India:482 U.S.:530	India: β = 0.14, U.S.: β = 0.03
Winkelmair & Jansen (2023) [51]	Interventional study	MBSR	FFMQ	2 items (Goal intention to eat more vegetarian meals)	12	91 (IG:65, CG:26)	t = -0.1
Winkelmair & Jansen (2024) [52]	Interventional study	MBSR	CHIME	Sustainable Consumption Behavior Nutrition Scale	8	137 (IG:66, CG:71)	Mean_Pre, IG_ = 3.59, SD_Pre, IG_ = 0.62, Mean_Post, IG_ = 3.63, SD_Post, IG_ = 0.55; Mean_Pre, CG_ = 3.59, SD_Pre, CG_ = 0.63, Mean_Post, CG_ = 3.59, SD_Post, IG_ = 0.60

*Abbreviations*: *BiNKA* Bildung für nachhaltigen Konsum durch Achtsamkeitstraining (Education for sustainable consumption through mindfulness training), *MBSR* Mindfulness-Based Stress Reduction, *sMBI* Sustainability-adapted mindfulness-based intervention, *MBI* Mindfulness-based Intervention, *PROMIS-29* Patient-Reported Outcomes Measurement Information System, *CHIME-A* Comprehensive Inventory of Mindfulness Experiences-Adolescents, *CHIME* Comprehensive Inventory of Mindfulness Experiences, *FFMQ* Five Facet Mindfulness Questionnaire, *PEB* Pro-environmental behavior, *SCB* Sustainable consumption behavior

The hand-search of relevant sources in the identified articles for inclusion revealed another four publications that were screened for eligibility. Of these, two were excluded from the final analysis as they assessed PEB generally and did not report nutrition-specific items.

The publication date of the studies was heavily concentrated in recent years, with all studies published between 2018 and 2025, reflecting the growing interest in this area. Of these studies, seven were intervention studies and six were observational studies. The majority of cases involved the assessment of mindfulness using the CHIME (*n* = 7) or FFMQ (*n* = 4) scales. Most studies were conducted in Europe (*n* = 8). Additionally, studies were conducted in North America, more specifically, the U.S. (*n* = 2) and in India (*n* = 2).

### Summary of main findings in the meta-analysis

**Fig. 2:**
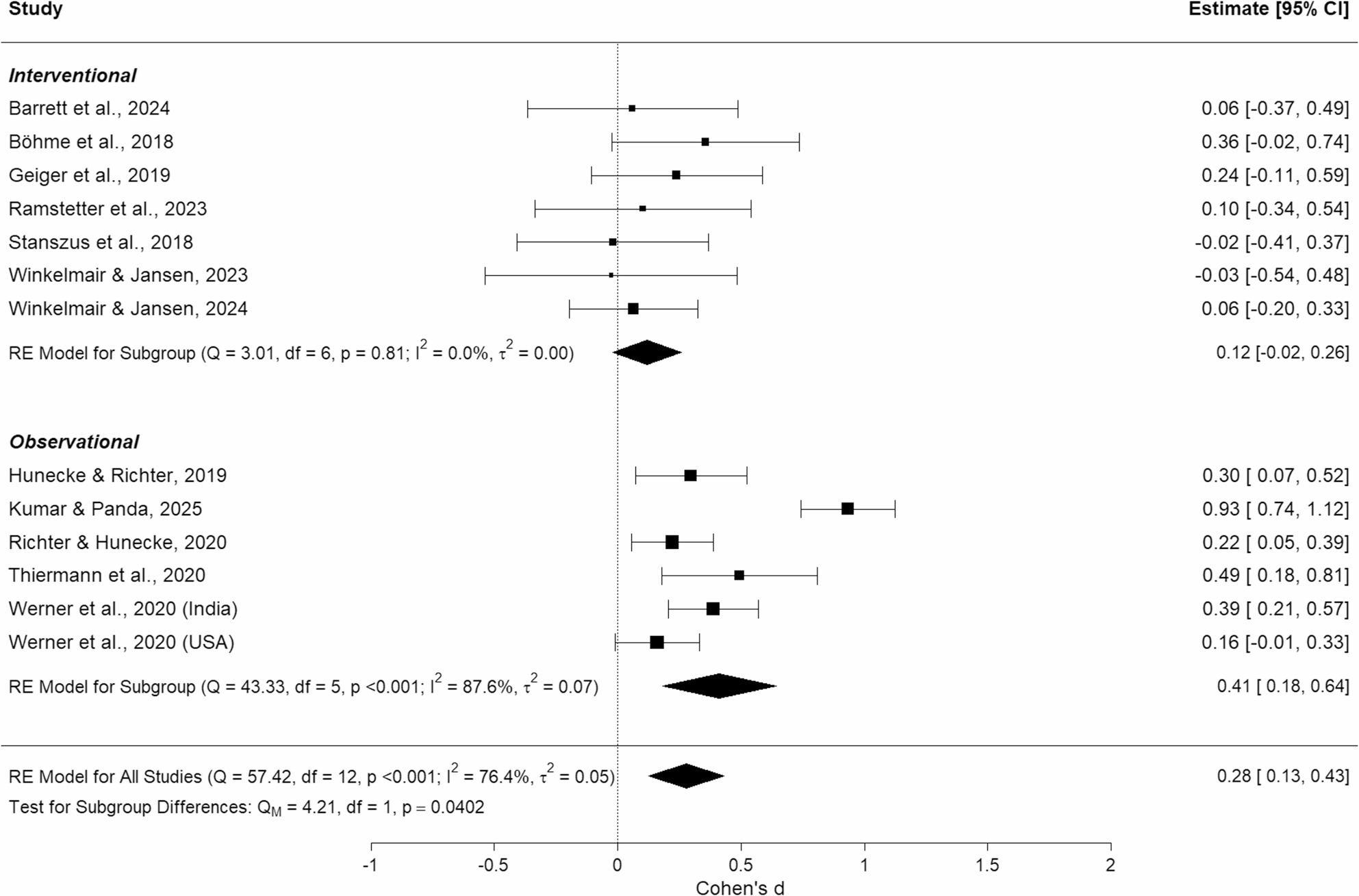
Forest plot of standardized effects (Cohen’s d) between mindfulness and sustainable dietary behavior. The analysis was stratified by study design (intervention vs. observational). Each horizontal line represents the 95% confidence interval for an individual study’s effect estimate. Intervention studies yielded more heterogeneous and mostly non-significant effects, whereas observational studies showed generally stronger and more consistent positive associations. The diamond shapes represent the estimated pooled effects from random-effects models for each subgroup and overall. Notable outliers [46] indicate residual heterogeneity within subgroups. Although observational studies appear to report larger effect sizes, the test for subgroup differences did not reach statistical significance, suggesting that study design may not robustly moderate the relationship

The overall effect of mindfulness on sustainable diets, as estimated by the random-effects model, was small but significant (*d* = 0.28, *SE* = 0.08, *z* = 3.57, *p* < .001, 95% CI [0.13, 0.43], Fig. 2). Effect sizes exhibited considerable variation, ranging from negative (e.g., Stanszus et al. [48]: *d* = -0.02, 95% CI [-0.41, 0.37]) to large positive values (e.g., Kumar & Panda [46]: *d* = 0.93, 95% CI [0.74, 1.12]). Several studies showed statistically significant positive effects of mindfulness on sustainable diets, with confidence intervals that did not cross zero – such as Hunecke and Richter [17], Richter and Hunecke [20] and Kumar and Panda [46].

Since for the intervention studies, the pre-post correlation *ρ* was missing for the calculation of *SE*(*d*), the meta-analysis was repeated with different *ρ* values. The results, however, differed only minorly from the results reported here and can be found in Supplemental Table S3. For the subsequent analysis, a pre-post correlation of *ρ* = 0.7 was used [53, 54].

### Statistical results of the meta-analysis

To assess heterogeneity among the included studies, a *Q*-test was conducted, which was statistically significant, *Q* (12) = 57.42, *p* < .001, indicating substantial variability across studies. Furthermore, the I^2^ statistics revealed that 76.41% of the total variation was due to true heterogeneity rather than sampling error. This high I^2^ value suggests that the observed differences in effect sizes are largely attributable to real differences between the studies rather than random variation. In addition to the *Q-*test and the I^2^ statistic, the between-study variance was estimated using the REML method, yielding a τ^2^ value of 0.05 (*SE* = 0.03) and a corresponding standard deviation τ of 0.23. These values further underscore the presence of substantial heterogeneity across the included studies.

To examine potential publication bias, a funnel plot and Egger’s regression test for funnel plot asymmetry were conducted. The funnel plot displayed slight asymmetry (Supplemental Figure S4). Yet, the Egger’s test did not confirm this (*z* = -1.56, *p* = .119). The estimated effect size as standard error approaches zero was *b* = 0.59, with 95% CI [0.17, 1.01] and no indication of publication bias was identified. To further validate these results, the trim-and-fill method was applied to the funnel plot (Supplemental Figure S5) and suggested the presence of four potentially missing studies, which were imputed on the right side of the funnel plot.

Influence diagnostics including a leave-one-out sensitivity analysis were conducted to assess the robustness of the overall meta-analytic findings (Supplemental Figure S6). Depending on the omitted study, the estimated effect sizes ranged from *d* = 0.23, *SE* = 0.04, *p* < .001, 95% CI [0.15, 0.31] to *d* = 0.30, *SE* = 0.08, *p* < .001, 95% CI [0.14, 0.46]. The exclusion of the study by Kumar and Panda [46] resulted in the most substantial change in the model estimates, leading to a notably reduced effect size and heterogeneity (τ^2^ = 0.00, I^2^ = 11.05%, = 11.98). Across all other iterations, heterogeneity remained high, as indicated by I^2^ values between 76.09% ad 78.72%. o evaluate the robustness of the meta-analytic findings under alternative statistical assumptions, the analysis was repeated using the Hartung-Knapp-Sidik-Jonkman method. Based on the twelve studies and thirteen study records, the estimated average effect size was *d* = 0.28, *SE* = 0.07, *t* (12) = 3.75, *p* = .003, 95% CI [0.12, 0.44]. The estimated heterogeneity was *τ*^*2*^ = 0.05 (*SE* = 0.03), corresponding to an I^2^ value of 76.41%.

### Subgroup analyses of the meta-analysis

Due to the substantial heterogeneity observed across the included studies (I^2^ = 76.41%), subgroup analyses were performed (Table 2). Study design emerged as a relevant moderator, with observational studies yielding statistically significant associations, whereas interventional studies showed non-significant associations, together explaining 23.50% of between-study variance (Fig. 2). In contrast, questionnaire type did not significantly account for variability (Supplemental Figure S7). Studies using the CHIME questionnaire showed significant effects. Studies employing the FFMQ questionnaire also demonstrated significant effects; however, their effect sizes did not differ significantly from those observed in CHIME studies. Likewise, the single study using the PROMIS-29 questionnaire showed no significant difference in effect size compared to CHIME. Finally, study region significantly moderated effect sizes (Supplemental Figure S8). Studies conducted in Asia showed considerably larger effects compared to those from Europe and North America. Accordingly, the study region explained 52.89% of the heterogeneity, though residual variance remained.

**Table 2:** Results of subgroup tests

Stratification criteria	Number of included estimates	Effect estimate	95% CI	I^2^	*p*^difference^ (Cochran’s Q test)	*R* ^2^
Study design	13	0.28	0.13, 0.43	76.41%	< 0.001	23.50%
Interventional	7	0.12	-0.02, 0.26	0.00%		
Observational	6	0.41	0.18, 0.64	87.6%		
Mindfulness Questionnaire	13	0.28	0.13, 0.43	76.41%	< 0.001	0.00%
CHIME	8	0.34	0.12, 0.56	83.2%		
FFMQ	4	0.22	0.10, 0.34	0.00%		
PROMIS-29	1	0.06	-0.37, 0.49	0.00%		
Continent	13	0.28	0.13, 0.43	76.41%	< 0.001	52.89%
Asia	2	0.66	0.12, 1.20	94.0%		
Europe	9	0.22	0.13, 0.31	0.00%		
North America	2	0.15	-0.01, 0.31	0.00%		

The number of studies, the effect estimate of the meta-analysis of the subgroups, the 95% CI, the remaining I^2^, the result of Cochran’s Q-test, and the explained R^2^of the subgroup analysis are reported

### Facet-specific meta-analysis

Various studies reported multiple facets of mindfulness and their influence on dietary behavior, thus, a separate meta-analysis was conducted for each reported facet. This facet-specific analysis was based on other effect sizes than those included in the overall meta-analysis and therefore did not constitute a traditional subgroup analysis. Rather, it served to explore whether distinct dimensions of mindfulness differed in their association with the outcome. Four studies assessed the impact of different facets of mindfulness and their influence on sustainable food consumption. Three studies that assessed mindfulness based on the FFMQ were included in the final meta-analysis: Hunecke and Richter [17], Richter and Hunecke [20], and Winkelmaier and Jansen [51]. The remaining study was excluded from the meta-analysis due to the use of the CHIME instrument rather than the FFMQ to assess mindfulness.

A separate meta-analysis was conducted for each of the five mindfulness facets included in the FFMQ. The results showed that three facets – observing (*d* = 0.39, 95% CI [0.26, 0.52]), describing (*d* = 0.14, 95% CI [0.01, 0.27]), and non-reactivity (*d* = 0.17, 95% CI [0.04, 0.30]), were significantly positively associated with sustainable dietary behavior, with small to moderate effect sizes and narrow confidence intervals. Non-judging (*d* = 0.13, 95% CI [-0.01, 0.27]) and acting with awareness (*d* = 0.01, 95% CI [-0.12, 0.14]) revealed a non-significant association.

### Meta-analysis on meat consumption reduction

In a separate meta-analysis, the potential relationship between mindfulness and reduced meat consumption was examined including all studies that reported on meat reduction in the context of mindfulness. The included studies either examined the relationship between mindfulness and adherence to a vegetarian or plant-based diet or focused on individuals’ intentions to reduce meat consumption. This analysis comprised five studies that provided relevant data (Barrett et al. [44], Hunecke and Richter [17], Thiermann et al. [49], Werner et al. [50] and Winkelmair and Jansen [51]). As in the overall meta-analysis, the study by Hunecke and Richter [17] was included using an average score across the FFMQ facets. For Werner et al. [50] both samples from India and the U.S. were again included in the analysis. The random-effects meta-analysis yielded a small to moderate, statistically significant overall effect size of *d* = 0.25 (*SE* = 0.06, *z* = 3.92, *p* < .001, 95% CI [0.13, 0.38]), suggesting that mindfulness is positively associated with reduced meat consumption or vegetarian dietary intentions (Supplemental Figure S9). Heterogeneity across the six included studies was low to moderate (τ^2^ = 0.008, I^2^ = 31.05%) and not statistically sinificant (*Q* (5) = 7.48, *p* = .188), indicating that much of the variation across studies could be attributed to sampling error rather than systematic differences.

### Cross-country CO_2_ reduction potential based on dietary shifts

To reflect realistic behavioural patterns of meat consumption and thus potential for CO_2_ emission reductions, we report results for three dietary scenarios: the current baseline (BAU), a partial dietary shift among mindful consumers (REDUCTION), based on the observed association between mindfulness and reduced meat consumption in the meta-analysis; see Environmental Impact Scenarios), and the full adoption of the Eat‑Lancet diet (LANCET). This structure allows us to compare the expected environmental savings under an optimal transformation with those achievable through more plausible, population‑level changes in dietary behaviour.

The mean and SD of LCA results (CO2 equivalents per kilogram bone-free meat) of different meat types (beef, pork, poultry) across regions (Germany, UK, U.S.) substantial regional disparities (Supplemental Table S10).

**Fig. 3:**
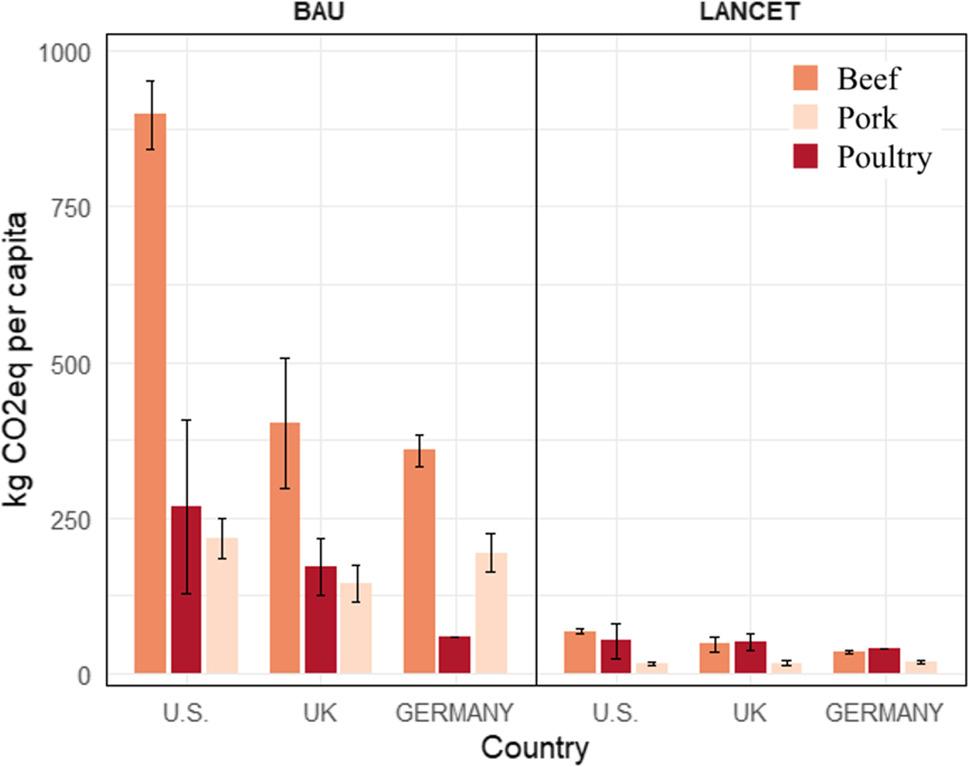
Comparison of emissions between the BAU and LANCET scenario per capita per country. Bars show mean emissions, arrows show standard deviation. Data basis for the emission values is LCA data from Clune et al. [36]. Current consumption of meat was based on meat consumption data reported by the FAO in 2022 (Our World in Data [55]. For the LANECT scenario, CO2 emissions per capita were based on Lancet recommendations for meat consumption in kg CO2-eq [56]. A total of 5.11 kg of red meat and 10.585 kg of poultry were taken as recommended values for meat consumption

Current emissions per capita varied significantly by country and meat type under both the BAU and the LANCET scenarios (Fig. 3). Under the BAU scenario, average annual emissions reached 1,382.35 kg CO_2_-eq in the U.S., compared to 612.45 kg CO_2_-eq in Germany and 719.68 kg CO_2_-eq in the UK. A notable factor contributing to the elevated emissions in the U.S. was the significantly higher per capita consumption of beef in the U.S. (53 kg) compared to Germany (16 kg) and the UK (35 kg).

Current emissions under the LANCET scenario were notably lower across all regions and meat types, which was to be expected given the substantially reduced quantities of meat consumed in this scenario.

Under the LANCET scenario, the U.S. maintained the highest average per capita emissions, amounting to 137.32 kg CO_2_-eq per person (Fig. 3). Since the allocation of red meat was based on current consumption patterns, the U.S. continued to demonstrate the highest rate of beef consumption, which was contributing to its particularly high emissions. For Germany, average per capita emissions decreased to 92.69 kg CO_2_-eq, while the UK exhibited a mean value of 117.16 kg CO_2_-eq under the LANCET scenario.

**Fig. 4:**
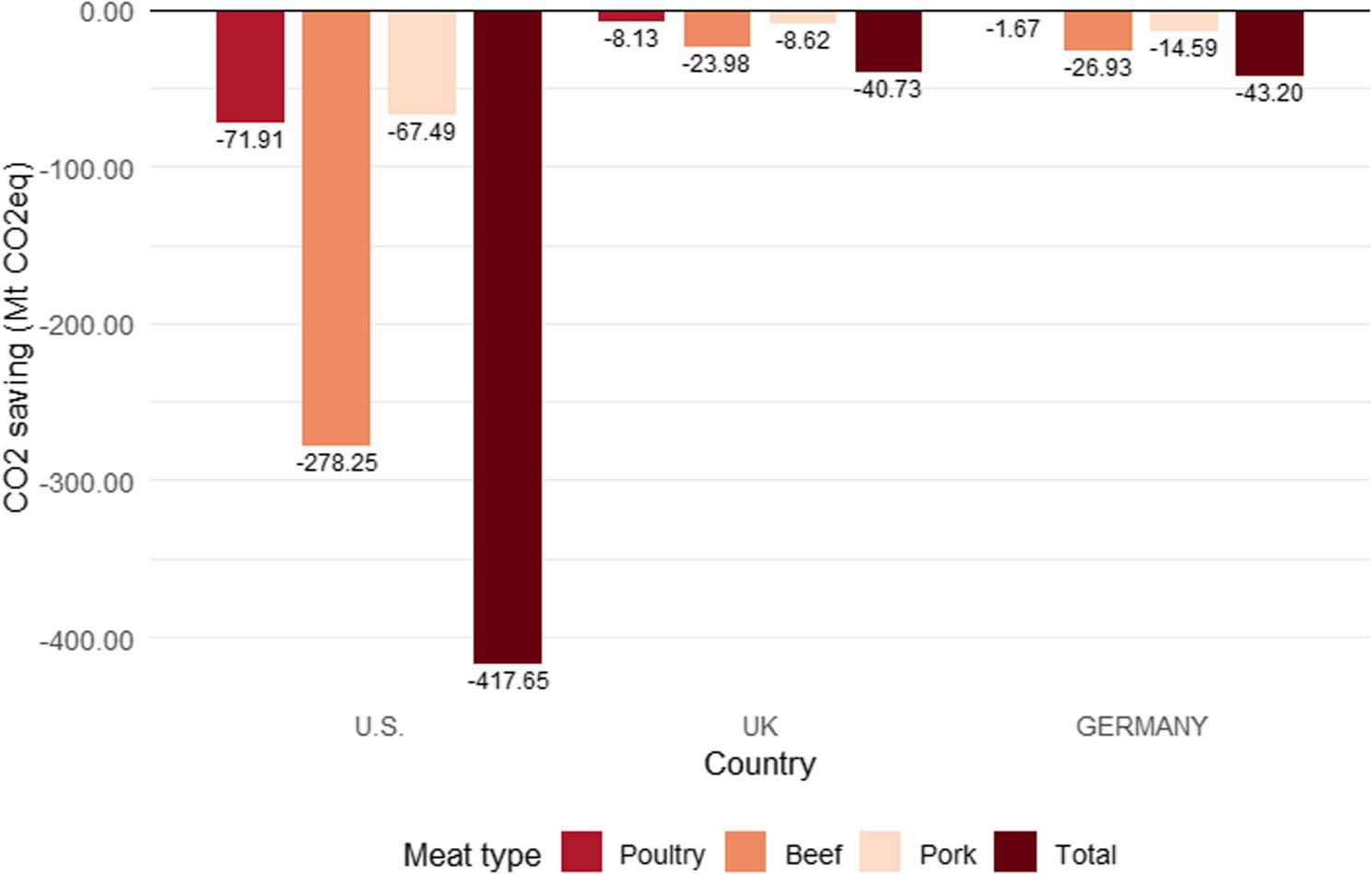
Model of the CO 2 reduction potential from adopting the EAT Lancet dietary guidelines across countries. Emission values were based on LCA data by Clune et al. [36]. The BAU scenario was based on current consumption levels of meat as reported by the FAO for 2022 (Our World in Data, 2024 [55]). For the LANECT scenario, CO2 emissions per capita were based on Lancet recommendations for meat consumption in kg CO2-Eq. [56]. A total of 5.11 kg of red meat and 10.585 kg of poultry were taken as recommended values for meat consumption. Inhabitant data for per country estimates was taken from official governmental websites of each region

A similar pattern could be observed when comparing potential CO_2_ savings across the three regions and meat types on country level (Fig. 4). The U.S. exhibited the highest CO_2_ reduction potential from a shift in meat consumption from the BAU scenario to the Lancet diet both across meat types and overall. This can partly be ascribed to the comparatively high baseline meat consumption in the U.S. and partly to its larger population size, which amplifies potential savings. Nevertheless, all three countries demonstrated substantial CO_2_ reduction potential if current meat consumption levels were aligned with the EAT Lancet recommendations.

### Scenario comparison of different reduction pathways

For future scenario comparison, a third scenario, the REDUCTION scenario, was introduced, reflecting a gradual dietary shift based on the survey data indicating the willingness to reduce meat consumption. To enable a clearer comparison, cumulative emissions to 2040 were calculated for all three scenarios and regions, based on each country’s average annual emissions. Under the BAU scenario, Germany emitted an average of 50.91 Mt CO_2_-eq of meat-related emissions per year, the UK 48.65 Mt CO_2_-eq, and the U.S. 463.71 Mt CO_2_-eq, assuming population of 83,118,501 million people in Germany, 67,596,000 in the UK, and 335,453,105 in the U.S. respectively in 2022 [37–39]. Conversely, the LANCET scenario resulted in significantly lower average annual emissions: 7.70 Mt CO_2_-eq meat-related emissions for Germany, 7.92 Mt CO_2_-eq for the UK, and 46.06 Mt CO2-eq for the U.S. The REDUCTION scenario ranged in the middle with 39.83 Mt CO_2_-eq yearly meat-related emissions in Germany, 28.93 Mt CO_2_-eq in the UK and 349.80 Mt CO_2_-eq in the U.S.

**Fig. 5:**
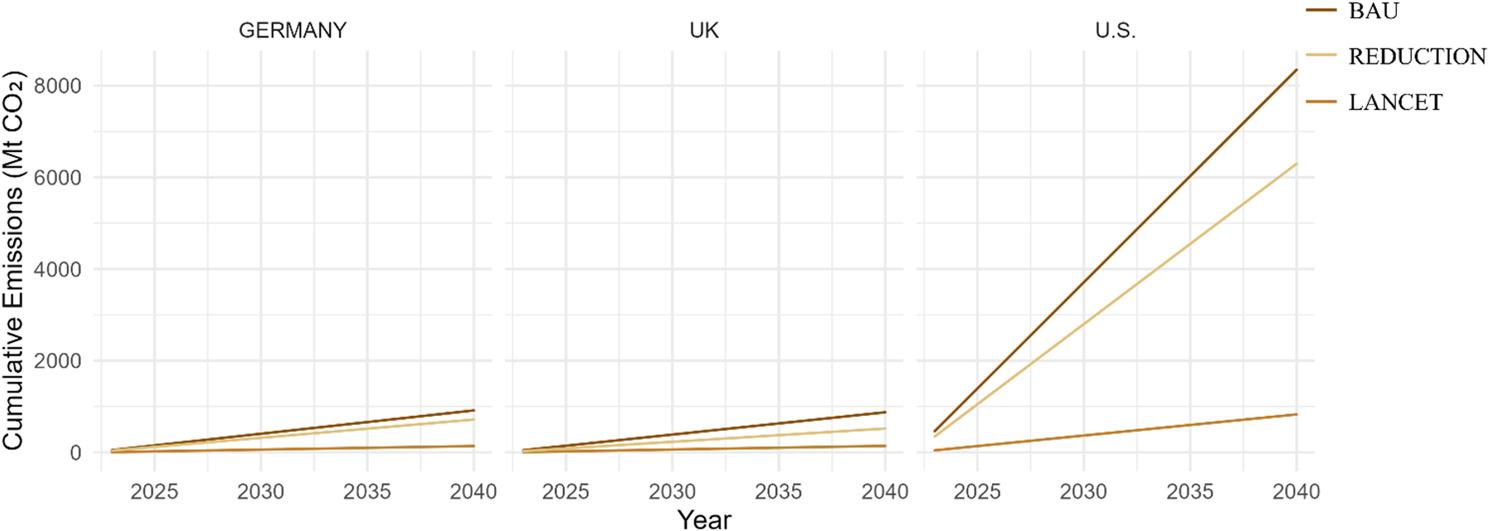
Cumulative emissions until 2040 for the different scenarios. A linear consumption pathway was assumed for the purpose of scenario comparison. The BAU scenario was based on current per capita meat consumption as reported by the FAO (Our World in Data, 2024 [55]). The number of inhabitants for emission calculations per country was based on inhabitant data as reported by the official government websites of Germany [37], the UK [39] and the U.S. [38]. Emissions for the LANCET Scenario were based on the recommendations by the EAT-Lancet commission, revealing allowable meat consumption of 5.11 kg of red meat and 10.585 kg of poultry per year [56]. The REDUCTION scenario was based on population-level survey data indicating the share of individuals who reported a willingness to reduce meat consumption [40–43]. For this scenario, the model assumed that part of the population continued to eat according to the BAU scenario, while another proportion of the population reduced meat intake to the levels prescribed by the Lancet diet

To enable scenario comparisons of future emission-reduction potential, a linear consumption trajectory was assumed. This approach distributes emission reductions from dietary changes evenly across all years through 2040 (Fig. 5). The data clearly demonstrate the considerable mitigation potential in all regions achievable with adopting either the REDUCTION or the LANCET pathway by 2040. The exact emission reduction potentials are summarized in Table 3.

**Table 3:** CO_2_ Emission savings under the different scenarios from 2023 until 2030, 2035 and 2040

Region	Until 2030		Until 2035		Until 2040	
	REDUCTION Savings (Mt CO2-eq)	LANCET Savings (Mt CO2-eq)	REDUCTION Savings (Mt CO2-eq)	LANCET Savings (Mt CO2-eq)	REDUCTION Savings (Mt CO2-eq)	LANCET Savings (Mt CO2-eq)
Germany	88.6	345.6	143.9	561.6	199.3	777.6
UK	157.7	325.8	256.5	529.5	354.9	733.1
U.S.	911.3	3341.2	1480.9	5429.5	2050.5	7517.7

A case study was conducted for Germany to estimate its CO_2_ emission budget in light of national reduction targets set out in the Federal Climate Change Act. The budget was then compared to the potential savings from a dietary shift towards the Lancet diet.

In accordance with the reduction goal of a 65% decrease in emissions compared to 1990 levels, the emissions budget for Germany from 2023 to 2030 amounts to 4,155 Mt of CO_2_-eq. For the period from 2030 to 2040, the remaining budget is 2,935.1 Mt CO_2_-eq, resulting in a total budget of 7,090.2 Mt CO_2_-eq for the timeframe 2023–2040.

A comparison of the LANCET scenario with the BAU scenario revealed cumulative savings of 777.6 Mt CO_2_-eq over the period, while the REDUCTION Scenario enables savings of 199.3 Mt CO_2_-eq. This corresponds to approximately 11% and 2.8%, respectively, of Germany’s total emissions budget, underscoring the significant contribution a dietary transition could make toward achieving climate targets.

## Discussion

This meta-analysis synthesized evidence from twelve studies, including thirteen study records, published between 2018 and 2025 examining the association between mindfulness and sustainable dietary behavior. Overall, mindfulness was found to have a small but significant positive association with sustainable dietary behaviour (d = 0.28), though effect sizes varied considerably across studies. These findings are consistent with prior evidence. In their scoping review, Pompili and Carfora (2025) [32] identified mindfulness as positively associated with sustainable eating, particularly through greater consumption of organic, local, and seasonal foods, with only one study reporting it as a barrier. Thus, the present study extends this evidence by providing quantitative support for the notion that mindfulness, as a process of reflection and shared experience, can promote sustainable food consumption and climate change mitigation. Additionally, the scoping review did not examine meat consumption as a distinct category.

Observational studies generally reported statistically significant associations, whereas intervention studies did not, and study region emerged as a significant moderator, with larger effect sizes observed in Asian compared to European or North American samples. Facet-level analyses indicated that specific dimensions of mindfulness – such as observing, describing, and non-reactivity – were significantly linked to more sustainable dietary behaviors. Observational studies may capture longer-term mindfulness engagement, while short-term interventions may lack lasting impact [22]. However, the binary classification of study design explained only limited variance, as substantial methodological differences persisted in both groups.

The subgroup analysis on mindfulness questionnaires revealed no significant moderator effect as the use of different mindfulness scales did not explain heterogeneity. This suggests that despite methodological and conceptual differences, the instruments largely converge in capturing the construct of mindfulness.

Conversely, the subgroup analysis investigating study regions as a moderator, was able to explain a large share of heterogeneity (R^2^ = 52.89%), with the strongest effects observed in India and weaker effects in Europe and North America. These differences likely reflect cultural factors as Asian cultures are more deeply rooted in mindfulness-related traditions such as Buddhism [57]. Moreover, Indian consumers tend to demonstrate lower attachment to meat, understood as a positive emotional and cognitive bond toward meat consumption, whereas meat consumption is particularly high in the U.S., where attachment to meat is also strong [55, 58, 59]. The highly influential study by Kumar and Panda (2025) [46], conducted among Indian Generation Z college students, further amplified the regional effect, highlighting the role of cultural and demographic context in shaping outcomes. This age cohort has been found to be more open to reducing meat consumption and exploring plant-based alternatives while higher educational attainment has also been associated with a greater openness to plant-based diets [60, 61].

The exploratory facet-level analysis suggested differential effects of mindfulness dimensions on sustainable nutrition. Observing showed the strongest positive association, aligning with prior findings that heightened attention to external stimuli may foster pro-environmental behavior [14]. Non-reactivity was also positively linked, supporting its role in disrupting automatic, environmentally harmful habits [14]. Describing unexpectedly emerged as a significant predictor, which may reflect enhanced awareness of internal cues relevant for mindful and sustainable eating [11, 62]. Non-judging showed a small, non-significant positive effect, consistent with mixed evidence on whether acceptance without evaluation facilitates or hinders behavioral change [14, 17]. Finally, acting with awareness was only marginally and non-significantly related to sustainable diets. Overall, these findings highlight that not all mindfulness facets contribute equally, underscoring the need for further differentiated research.

The additional meta-analysis on meat reduction revealed a small but significant positive association with mindfulness (d = 0.25). Despite the broad spectrum of measured effect sizes included in the analysis, a positive association between mindfulness and meat reduction was observed and heterogeneity was moderate (I^2^ = 31.05%). These findings indicate that mindfulness may represent a promising component in promoting more sustainable behaviors that remain within planetary boundaries [29, 63].

Meat consumption is a major contributor to dietary-related GHG emissions [64]. Based on the observed link between mindfulness and reduced meat intake, an additional analysis estimated potential emission reductions under different dietary scenarios (see “Environmental Impact Scenarios”).

Other studies have also found the association of mindful eating with healthier, more plant‑based diets and lower consumption of animal products (e.g [65]), however do not estimate CO₂ emissions and any environmental benefit via separate environmental‑impact modeling and thus reduction potential is only inferred indirectly from the dietary shifts described.

Our results suggest that significant reductions in CO_2_ emissions could be realized through broader adoption of a predominantly plant-based diet. The scenario analysis includes several limitations inherent, making careful interpretation of the mean values essential.

The BAU scenario assumes constant dietary patterns and thus overlooks recent fluctuations, such as Germany’s gradual decline in meat consumption and its slight increase in 2024 [55, 66]. However, such simplifications are common and necessary in scenario analyses to model complex systems [67].

The EAT-Lancet diet framework from 2019 was used as the reference scenario, as it provides the first scientifically derived, quantifiable framework for sustainable meat consumption [56]. While offering clear dietary boundaries, its recommendations are highly ambitious compared to current patterns – for example, Germany’s 2022 red meat consumption of 53 kg per capita would need to decline by over 90% to meet the Lancet target of 5.11 kg. Nonetheless, exploring options that extend beyond current operational and conceptual boundaries is a key element of scenario analyses and one of the factors how scenarios can contribute to the realization of desirable futures [67].

In the REDUCTION scenario, survey data were used to estimate the share of individuals willing to reduce meat consumption, with their emissions modeled according to the EAT-Lancet diet and the remainder kept at BAU. However, due to the attitude–behavior gap, such stated willingness does not necessarily reflect actual reductions to Lancet levels.

The scenario analysis showed that meat-related emissions remain high in Germany, the UK, and the U.S., with current diets far from sustainability targets. Even partial adoption of the Lancet diet yielded notable CO_2_ reductions, though emissions are only one aspect of sustainable nutrition, which also includes environmental and health dimensions. Reduced red meat intake has been linked to lower risks of cancer [68], cardiovascular disease [69], and type 2 diabetes [70]. Overall, current levels of meat consumption are incompatible with the goals of sustainable nutrition and continue to strain planetary boundaries [7]. Given the considerable gap between current dietary behaviors and sustainability targets, multiple strategies should be explored and implemented to move closer to an optimal dietary model. Mindfulness should not be overlooked as a potentially supportive factor, and further research is warranted to examine its role in promoting more sustainable eating habits.

To contextualize the scenario results and derive policy implications, an additional calculation estimated the share of Germany’s emission budget until 2040 that could be saved through a dietary shift to the EAT-Lancet diet. If the entire population adopted this diet, savings would equal about 11% of the national budget, while the more conservative REDUCTION scenario still achieved around 2.8%. These results underscore dietary change as an important lever for climate policy and support the need for targeted programs and awareness campaigns to address the impact of meat consumption and promote its reduction.

Despite the valuable findings, several limitations must be acknowledged. Comparability between studies was limited, as some measures were adapted or shortened, study foci ranged from general populations to specific groups (e.g. Gen Z), sample sizes were often small, and regional coverage uneven. The operationalization of sustainable diets also varied, with studies assessing organic food consumption, vegetarian diets, or meat reduction. These operationalizations are not directly comparable as meat and dairy are consistently linked to high emissions in contrast to the still debated trade-offs of organic farming. Methodological heterogeneity further constrained conclusions, with differing control conditions in interventional studies and reliance on self-reported measurements that may be biased as individuals without meditation experience may misinterpret questionnaire items. Additional limitations arose from methodological assumptions, including potential bias from standardizing effect sizes and estimating missing pre-post correlations. Finally, the analysis focused solely on CO_2_ reductions from meat avoidance, neglecting impacts of protein substitutes and it relied on the ambitious assumption of the EAT Lancet diet, limiting practical feasibility.

Future research should conduct further moderator analyses to examine whether cultural or demographic factors (e.g. age, sex, education) can explain heterogeneity with regards to mindfulness and sustainable dietary behaviors. Additionally, Connectedness to Nature would be an interesting moderator to be explored. It would also be valuable to differentiate between specific mindfulness facets, including those captured by the CHIME questionnaire, as this study provided indications that some facets facilitate sustainable eating, while others hinder it. Moreover, future studies should examine distinct dimensions of sustainable diets, such as meat reduction, and preferences for organic, seasonal, or local foods, rather than treating sustainable diets as a single construct, in order to gain more nuanced insights; including with regard to translating these insights into the potential for reducing CO2 emissions in food production and transportation.

## Conclusions

This study shows that mindfulness has the potential to support shifts toward more sustainable diets, including reduced meat consumption, thereby contributing to climate mitigation and public health. At the same time, it is important to acknowledge that the most substantial reductions in climate change impacts require systemic policy action and meaningful mitigation efforts by governments and major industry actors (e.g., food system), while individual dietary change represents one of the most effective climate-relevant actions at the citizen level. While the effects observed are small and vary across contexts, they highlight mindfulness as an underexplored factor in dietary transition strategies. By fostering awareness, compassion, and reflection on daily food choices, mindfulness can help bridge the gap between attitudes and sustainable behavior. To fully realize this potential, future work should refine intervention approaches, address cultural and methodological heterogeneity, and explore how mindfulness-based programs can be integrated into broader policies and practices promoting planetary health.

## Supplementary Information

Supplementary Material 1.

## Data Availability

The datasets used and/or analysed during the current study are available from the corresponding author on reasonable request.
