# Supplementary material for: Mindfulness and sustainable diets: a meta-analysis and CO2 emission savings scenarios
Source: Nutr J. 2026 Jul 6;25:73. doi: 10.1186/s12937-026-01356-0 (PMC13339993; doi:10.1186/s12937-026-01356-0)
Supplement: Supplementary file 1 — Supplementary Material 1. [file 12937_2026_1356_MOESM1_ESM.docx]

**Supplementary Materials**

**Mindfulness and Sustainable Diets: A Meta-Analysis and CO_2_ Emission Savings Scenarios**

Authors: Anna Kosteletzky^1^, Stephanie Margarete Thomas^1,2^, Carmen Jochem^3^

^1^ Department of Biogeography, University of Bayreuth, Bayreuth, Germany

^2^ Bayreuth Center of Ecology and Environmental Research BayCEER, Bayreuth, Germany

^3^ Chair of Planetary & Public Health, University of Bayreuth, Bayreuth, German

**Supplemental Table S1: PRISMA**

| **Section and Topic** | **Item #** | **Checklist item** | **Location where item is reported** |
| --- | --- | --- | --- |
| **TITLE** | | |  |
| Title | 1 | Identify the report as a systematic review. | Title Page |
| **ABSTRACT** | | |  |
| Abstract | 2 | See the PRISMA 2020 for Abstracts checklist. | P. 2-3 |
| **INTRODUCTION** | | |  |
| Rationale | 3 | Describe the rationale for the review in the context of existing knowledge. | P. 4-5 |
| Objectives | 4 | Provide an explicit statement of the objective(s) or question(s) the review addresses. | P. 5 |
| **METHODS** | | |  |
| Eligibility criteria | 5 | Specify the inclusion and exclusion criteria for the review and how studies were grouped for the syntheses. | P. 5 |
| Information sources | 6 | Specify all databases, registers, websites, organisations, reference lists and other sources searched or consulted to identify studies. Specify the date when each source was last searched or consulted. | P. 6 |
| Search strategy | 7 | Present the full search strategies for all databases, registers and websites, including any filters and limits used. | Supplemental Table S2 |
| Selection process | 8 | Specify the methods used to decide whether a study met the inclusion criteria of the review, including how many reviewers screened each record and each report retrieved, whether they worked independently, and if applicable, details of automation tools used in the process. | P. 6 |
| Data collection process | 9 | Specify the methods used to collect data from reports, including how many reviewers collected data from each report, whether they worked independently, any processes for obtaining or confirming data from study investigators, and if applicable, details of automation tools used in the process. | P. 6 |
| Data items | 10a | List and define all outcomes for which data were sought. Specify whether all results that were compatible with each outcome domain in each study were sought (e.g. for all measures, time points, analyses), and if not, the methods used to decide which results to collect. | P. 8 (Table 1) |
|  | 10b | List and define all other variables for which data were sought (e.g. participant and intervention characteristics, funding sources). Describe any assumptions made about any missing or unclear information. | P. 8-9 |
| Study risk of bias assessment | 11 | Specify the methods used to assess risk of bias in the included studies, including details of the tool(s) used, how many reviewers assessed each study and whether they worked independently, and if applicable, details of automation tools used in the process. | / |
| Effect measures | 12 | Specify for each outcome the effect measure(s) (e.g. risk ratio, mean difference) used in the synthesis or presentation of results. | P. 8 (Table 1) |
| Synthesis methods | 13a | Describe the processes used to decide which studies were eligible for each synthesis (e.g. tabulating the study intervention characteristics and comparing against the planned groups for each synthesis (item #5)). | P. 7 |
|  | 13b | Describe any methods required to prepare the data for presentation or synthesis, such as handling of missing summary statistics, or data conversions. | P. 8-9 |
|  | 13c | Describe any methods used to tabulate or visually display results of individual studies and syntheses. | P. 7 |
|  | 13d | Describe any methods used to synthesize results and provide a rationale for the choice(s). If meta-analysis was performed, describe the model(s), method(s) to identify the presence and extent of statistical heterogeneity, and software package(s) used. | P. 7 |
|  | 13e | Describe any methods used to explore possible causes of heterogeneity among study results (e.g. subgroup analysis, meta-regression). | P. 7 |
|  | 13f | Describe any sensitivity analyses conducted to assess robustness of the synthesized results. | P. 7 |
| Reporting bias assessment | 14 | Describe any methods used to assess risk of bias due to missing results in a synthesis (arising from reporting biases). | P. 7 |
| Certainty assessment | 15 | Describe any methods used to assess certainty (or confidence) in the body of evidence for an outcome. | / |
| **RESULTS** | | |  |
| Study selection | 16a | Describe the results of the search and selection process, from the number of records identified in the search to the number of studies included in the review, ideally using a flow diagram. | P. 8 (Figure 1) |
|  | 16b | Cite studies that might appear to meet the inclusion criteria, but which were excluded, and explain why they were excluded. | P. 8 (Figure 1) |
| Study characteristics | 17 | Cite each included study and present its characteristics. | P. 8 (Table 1) |
| Risk of bias in studies | 18 | Present assessments of risk of bias for each included study. | / |
| Results of individual studies | 19 | For all outcomes, present, for each study: (a) summary statistics for each group (where appropriate) and (b) an effect estimate and its precision (e.g. confidence/credible interval), ideally using structured tables or plots. | P. 8 (Figure 2), P. 10-11 (Table 2) |
| Results of syntheses | 20a | For each synthesis, briefly summarise the characteristics and risk of bias among contributing studies. | + |
|  | 20b | Present results of all statistical syntheses conducted. If meta-analysis was done, present for each the summary estimate and its precision (e.g. confidence/credible interval) and measures of statistical heterogeneity. If comparing groups, describe the direction of the effect. | P. 10 (Table 2) |
|  | 20c | Present results of all investigations of possible causes of heterogeneity among study results. | P. 10 |
|  | 20d | Present results of all sensitivity analyses conducted to assess the robustness of the synthesized results. | P. 9-10 |
| Reporting biases | 21 | Present assessments of risk of bias due to missing results (arising from reporting biases) for each synthesis assessed. | P. 9 |
| Certainty of evidence | 22 | Present assessments of certainty (or confidence) in the body of evidence for each outcome assessed. | / |
| **DISCUSSION** | | |  |
| Discussion | 23a | Provide a general interpretation of the results in the context of other evidence. | P. 15 |
|  | 23b | Discuss any limitations of the evidence included in the review. | P. 19 |
|  | 23c | Discuss any limitations of the review processes used. | P. 19 |
|  | 23d | Discuss implications of the results for practice, policy, and future research. | P. 19-20 |
| **OTHER INFORMATION** | | |  |
| Registration and protocol | 24a | Provide registration information for the review, including register name and registration number, or state that the review was not registered. | P. 5 |
|  | 24b | Indicate where the review protocol can be accessed, or state that a protocol was not prepared. | P. 5 |
|  | 24c | Describe and explain any amendments to information provided at registration or in the protocol. | / |
| Support | 25 | Describe sources of financial or non-financial support for the review, and the role of the funders or sponsors in the review. | P. 20 |
| Competing interests | 26 | Declare any competing interests of review authors. | P. 20 |
| Availability of data, code and other materials | 27 | Report which of the following are publicly available and where they can be found: template data collection forms; data extracted from included studies; data used for all analyses; analytic code; any other materials used in the review. | P. 20 |

**Supplemental Table S2: Search term**

Search Term PubMed

("mindful*"[Title/Abstract] OR "MBSR"[Title/Abstract] OR "meditat*"[Title/Abstract] OR "stress reduction"[Title/Abstract] OR "loving-kindness"[Title/Abstract] OR "metta"[Title/Abstract] OR "heartful*"[Title/Abstract]) AND ("sustainab*"[Title/Abstract] OR "environment*"[Title/Abstract] OR "ecologic*"[Title/Abstract] OR "ethic*"[Title/Abstract] OR "green*"[Title/Abstract] OR "natur*"[Title/Abstract] OR "carbon footprint"[Title/Abstract]) AND ("eating"[Title/Abstract] OR "food"[Title/Abstract] OR ("nutritional status"[MeSH Terms] OR "nutritional sciences"[MeSH Terms]) OR "nutrition"[Title/Abstract] OR "diet"[MeSH Terms] OR "diet*"[Title/Abstract] OR "plant-based"[Title/Abstract] OR "vegetar*"[Title/Abstract] OR "vegan"[Title/Abstract] OR "eat"[Title/Abstract])

Search Term Web of Science

(TI=("mindful*" OR "MBSR" OR "meditat*" OR "stress reduction" OR "loving-kindness" OR "metta" OR "heartful*") OR AB=("mindful*" OR "MBSR" OR "meditat*" OR "stress reduction" OR "loving-kindness" OR "metta" OR "heartful*"))AND (TI=("sustainab*" OR "environment*" OR "ecologic*" OR "ethic*" OR "green*" OR "natur*" OR "carbon footprint") OR AB=("sustainab*" OR "environment*" OR "ecologic*" OR "ethic*" OR "green*" OR "natur*" OR "carbon footprint")) AND (TI=("eating" OR "food" OR "nutrition" OR "nutritional status" OR "nutritional sciences" OR "diet" OR "diet*" OR "plant-based" OR "vegetar*" OR "vegan" OR "eat") OR AB=("eating" OR "food" OR "nutrition" OR "nutritional status" OR "nutritional sciences" OR "diet" OR "diet*" OR "plant-based" OR "vegetar*" OR "vegan" OR "eat")

**Supplemental Table S3: pre-post findings**

| **Assumed pre-post correlation** | **Effect size** | **95 % CI** | **τ^2^** | **I^2^** | **Q-Test** |
| --- | --- | --- | --- | --- | --- |
| ρ = 0.0 | 0.30 | [0.14, 0.46] | 0.05 | 73.57 % | Q(12) = 51.07; p < .001 |
| ρ = 0.5 | 0.29 | [0.13, 0.44] | 0.05 | 74.79 % | Q(12) = 53.59; p < .001 |
| ρ = 0.9 | 0.27 | [0.12, 0.41] | 0.06 | 81.59 % | Q(12) = 70.64; p < .001 |

**Supplemental Figure S4: Funnel plot**


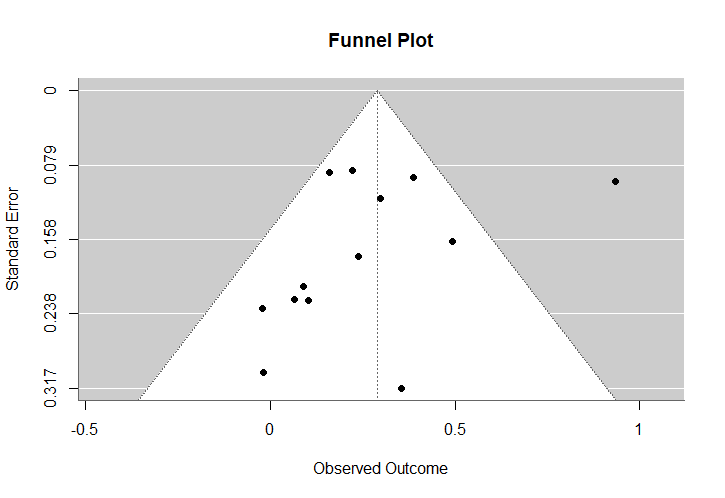


*Supplemental Figure S4. Funnel Plot assessing publication bias. The funnel plot exhibits slight asymmetric. The associated Egger’s test was insignificant; thus, no publication bias could be detected.*

**Supplemental Figure S5: Trim-and-Fill method applied to the funnel plot**


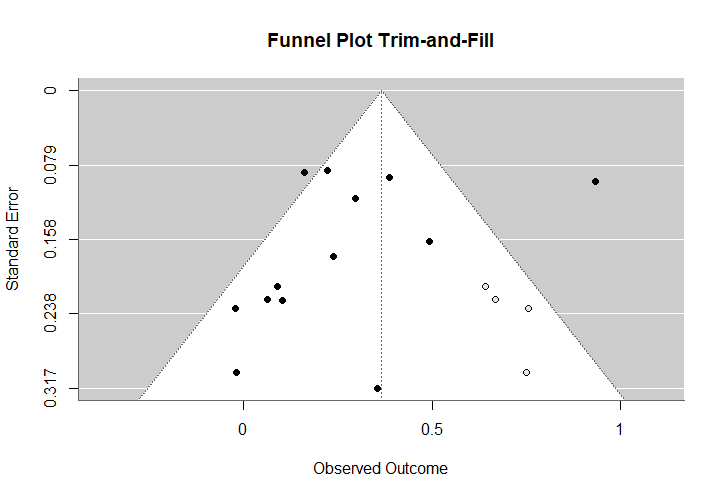


*Supplemental Figure S5. Trim-and-fill method applied to the funnel plot. Observed studies are shown as black circles; imputed missing studies are shown as white circles. Studies are missing on the right side of the funnel plot, indicating that studies with higher effect sizes are potentially missing.*

**Supplemental Figure S6: Influence diagnostics of the meta-analytical model**


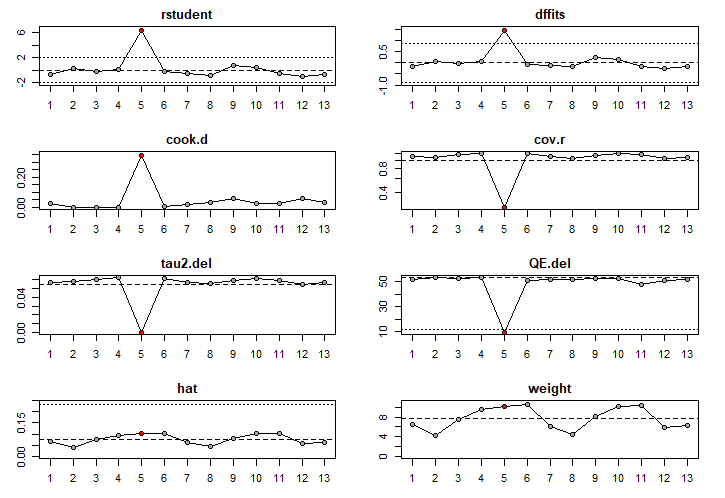


*Supplemental Figure S6. Visual results of the influence diagnostics for the meta-analytic model. Plots display measures of outlier and influence detection for each study: studentized residuals (rstudent), DFFITS, Cook’s distance (cook.d), covariance ratio (cov.r), changes in between-study variance (tau².del) and Q-statistic (QE.del) after study removal, leverage (hat), and study weights. Study 5 (the study by Kumar and Panda (2025)) stands out across multiple indicators (marked red), showing a high studentized residual (> 6), the highest DFFITS and Cook’s distance, a marked reduction in tau² and QE when excluded, a low COVRATIO, and a high weight—suggesting a strong influence on the model and contribution to heterogeneity. Study 1: Barrett et al. (2024); Study 2: Böhme et al. (2018); Study 3: Geiger et al. (2019); Study 4: Hunecke & Richter (2019); Study 5: Kumar & Panda (2025); Study 6: Ramstetter et al. (2023); Study 7: Richter & Hunecke (2020); Study 8: Stanszus et al. (2018); Study 9: Thiermann et al. (2020); Study 10: Werner et al. (2020): India; Study 11: Werner et al. (2020): USA; Study 12: Winkelmair & Jansen (2023); Study 13: Winkelmair & Jansen (2024)*

**Supplemental Figure S7: Forest Plot of subgroup analysis questionnaire type**
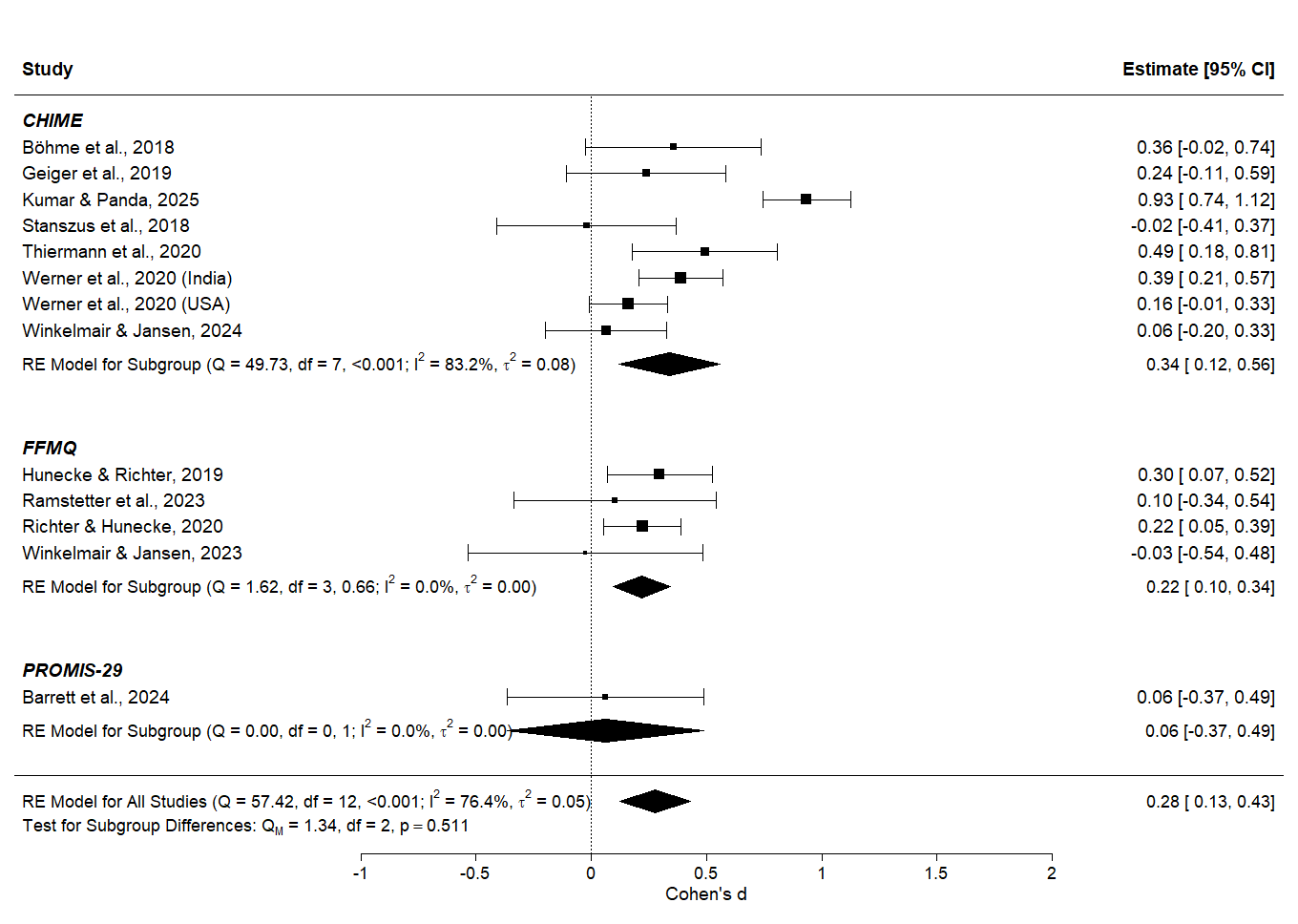


*Supplemental Figure S7. Forest plot showing standardized effect sizes (Cohen’s d) for the relationship between mindfulness and sustainable dietary behavior, stratified by type of mindfulness questionnaire (CHIME, FFMQ, PROMIS-29). Each horizontal line represents the 95% confidence interval for an individual study’s effect estimate. For each study, the calculated Cohen’s d values and corresponding 95% CIs are reported. The diamond shapes reflect pooled effect estimates from random-effects models for each questionnaire subgroup and overall. While some studies (e.g., Kumar & Panda, 2025) deviate notably from subgroup trends, overall differences between questionnaire types were not statistically significant, suggesting that the type of instrument used to assess mindfulness did not meaningfully moderate the observed associations.*

**Supplemental Figure S8: Forest Plot of subgroup analysis continent**


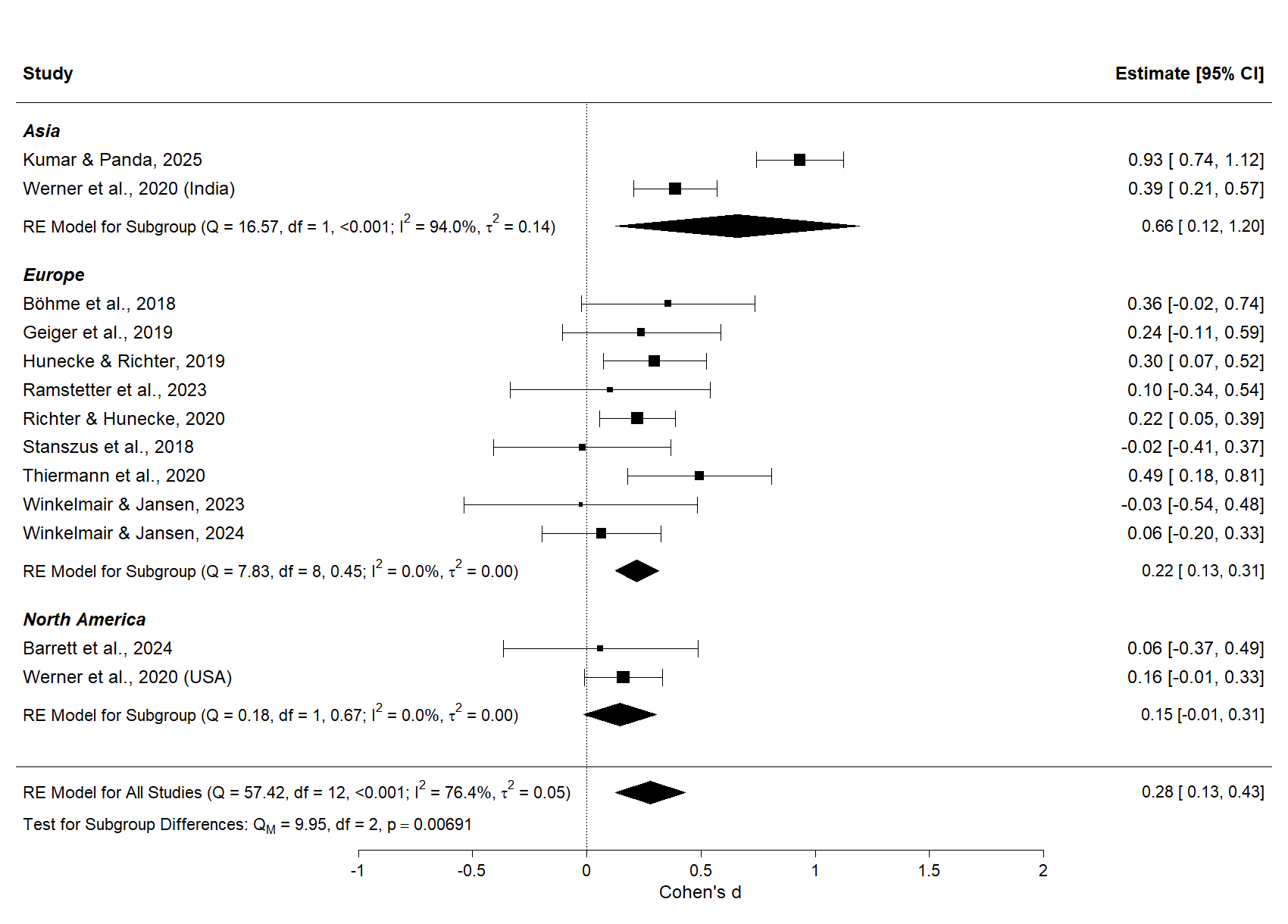


*Supplemental Figure S8. Forest plot showing standardized effect sizes (Cohen’s d) for the relationship between mindfulness and sustainable dietary behavior, stratified by study region (Asia, Europe, North America). Each horizontal line represents the 95% confidence interval for an individual study’s effect estimate. For each study, the calculated Cohen’s d values and corresponding 95% CIs are reported. Diamond shapes indicate pooled effect sizes from random-effects models for each region and across all studies. Notable deviations from subgroup estimates – such as the particularly strong effect reported by Kumar & Panda (2025) – highlight remaining heterogeneity within regions. The analysis supports the finding that study region significantly moderated effect sizes in the meta-analysis.*

**Supplemental Figure S9: Forest Plot of the meta-analysis on meat consumption reduction**


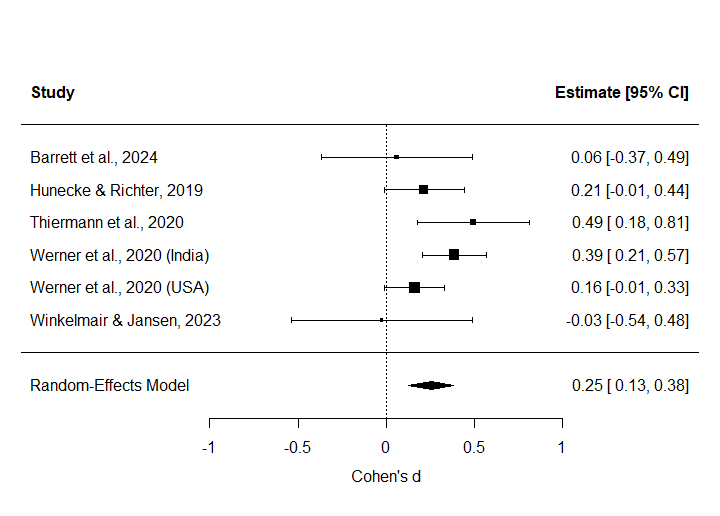


*Supplemental Figure S9. Forest Plot showing the association between mindfulness and a reduction in meat consumption based on the calculated standardized mean difference (Cohen’s d). A random effects model was used to account for high between-study heterogeneity. All effect sizes extracted from the studies were converted into Cohen’s d for comparability. Overall, a small but significant association was identified (d=0.25, CI [0.13, 0.38]. The position of the squares indicates the estimated effect size for each study; the bars show the standard error of Cohen’s d. The size of the squares reflects the weight of the study in the meta-analysis. Werner et al. (2020) is included twice since the paper evaluated two independent samples in different countries. For each study, the calculated effect size as well as the 95% CI are reported.*

**Supplemental Table S10: Life Cycle Assessment results of different meat types across regions**

| Region | Food type | Mean (CO_2_ eq / kg of bone-free meat) | SD (CO_2_ eq / kg of bone-free meat) | N |
| --- | --- | --- | --- | --- |
| Germany | Beef | 25.6 | 1.86 | 2 |
| Germany | Pork | 4.98 | 0.785 | 4 |
| Germany | Poultry | 3.72 | NA | 1 |
| UK | Beef | 23.7 | 6.17 | 17 |
| UK | Pork | 5.57 | 1.13 | 13 |
| UK | Poultry | 4.92 | 1.30 | 20 |
| U.S. | Beef | 23.6 | 1.42 | 4 |
| U.S. | Pork | 7.25 | 1.10 | 7 |
| U.S. | Poultry | 5.05 | 2.64 | 6 |
